# Serum ammonia variation predicts mortality in patients with hepatitis B virus-related acute-on-chronic liver failure

**DOI:** 10.3389/fmicb.2023.1282106

**Published:** 2023-12-04

**Authors:** Yi-Jing Cai, Jia-Jia Dong, Rui-Cong Chen, Qian-Qian Xiao, Xu-Mei Li, De-Yuan Chen, Chao Cai, Xiu-Li Lin, Ke-Qing Shi, Ming-Qin Lu

**Affiliations:** ^1^Department of Infectious Diseases, The First Affiliated Hospital of Wenzhou Medical University, Wenzhou, Zhejiang, China; ^2^Translational Medicine Laboratory, The First Affiliated Hospital of Wenzhou Medical University, Wenzhou, Zhejiang, China; ^3^Department of Ultrasonography, The First Affiliated Hospital of Wenzhou Medical University, Wenzhou, Zhejiang, China; ^4^Department of Pathology, The First Affiliated Hospital of Wenzhou Medical University, Wenzhou, Zhejiang, China

**Keywords:** ammonia, AMM, hepatitis B virus, acute-on-chronic liver failure, hepatic encephalopathy (HE), hepatitis B virus-related acute-on-chronic liver failure (HBV-ACLF), serum ammonia/reference laboratory upper limit of normal for ammonia (AMM-ULN), variation

## Abstract

**Background:**

Hyperammonemia is critical to the development of hepatic encephalopathy (HE) and is associated with mortality in end-stage liver disease. This study investigated the clinical value of ammonia variation in hepatitis B virus-related acute-on-chronic liver failure (HBV-ACLF) patients.

**Methods:**

A total of 276 patients with HBV-ACLF were retrospectively recruited. Patients' ammonia levels were serially documented. Baseline ammonia, Peak ammonia (highest level), and Trough ammonia (lowest level) were particularly corrected to the upper limit of normal (AMM-ULN). The primary endpoint was 28-day mortality.

**Results:**

The 28-day, 3-month, and 12-month mortality rates were 19.2, 25.7, and 28.2%, respectively. A total of 51 (18.4%) patients had overt HE (grade 2/3/4). Peak AMM-ULN was significantly higher in patients with overt HE and non-survivors compared with their counterparts (*P* < 0.001). Following adjustment for significant confounders, high Peak AMM-ULN was an independent predictor of overt HE (hazard ratio, 1.031, *P* < 0.001) and 28-day mortality (hazard ratio, 1.026, *P* < 0.001). The cut-off of Peak AMM-ULN was 1.8, determined by using the X-tile. Patients with Peak AMM-ULN appearing on days 1–3 after admission had a higher proportion of overt HE and mortality compared to other groups. Patients with decreased ammonia levels within 7 days had better clinical outcomes than those with increased ammonia.

**Conclusion:**

Serum Peak ammonia was independently associated with overt HE and mortality in HBV-ACLF patients. Serial serum ammonia may have prognostic value.

## 1 Introduction

Hepatic encephalopathy (HE) is a frequent and severe complication of end-stage liver diseases and is associated with mortality. Hyperammonemia has been proven to be central in the pathophysiology of hepatic encephalopathy as well as being associated with intracranial hypertension leading to death. In the setting of ACLF, there is an increased risk of brain edema and mortality with hyperammonemia, and its resolution is associated with improved outcomes (Bhatia et al., 2006; Bernal et al., 2007). The mechanism of hyperammonemia affecting end-stage liver diseases is mostly recognized for its role in HE. However, previous studies also showed that hyperammonemia can provoke immune dysfunction and compromised neutrophil function (Shawcross et al., 2008), predisposing patients to infection (Shalimar et al., 2017), exacerbate sarcopenia (Kumar et al., 2017), and even exert direct hepatoxicity (Jia et al., 2014).

Acute-on-chronic liver failure (ACLF) is a syndrome characterized by an acute deterioration of chronic liver disease complicated with other organ failures in association with high mortality. There is disagreement regarding ACLF diagnostic criteria between Eastern and Western countries because of etiology, heterogeneity, and different ethnicities. The definitions proposed by the Asian-Pacific Association for the Study of the Liver (APASL) and the European Association for the Study of the Liver (EASL) are widely accepted. The APASL defines ACLF as jaundice [total bilirubin (TB) ≥5 mg/dl] and coagulopathy [international normalized ratio (INR) ≥1.5 or prothrombin activity (PTA) <40%], complicated within 4 weeks by ascites and/or HE in 2019 (Sarin et al., 2019). HBV infection is the major etiology of liver disease in the Asia-Pacific region and can progress into ACLF in patients with HBV reactivation.

Elevated serum ammonia has been reported in ACLF patients with HE. Studies have highlighted that high ammonia levels on admission are an independent factor in predicting mortality in patients with ACLF (Zhang et al., 2020; Chiriac et al., 2021). Furthermore, Shalimar et al. (2020) reported that patients with persistent hyperammonemia during the first 3 days of hospitalization developed a higher proportion of organ failures and had higher 28-day mortality than patients with acute decompensation and ACLF. Another study performed by Sawhney et al. (2016) showed ammonia on admission did not correlate with survival. However, failing to decrease ammonia levels in patients with ACLF increases the risk of death (Sawhney et al., 2016). Despite these known correlations, the role of ammonia level, especially its dynamic changes in the APASL definition of HBV-ACLF, remains to be elucidated. Therefore, the present study was conducted by analyzing ammonia levels to determine whether ammonia variation during hospitalization is associated with HE and mortality in an APASL definition of the HBV-ACLF cohort.

## 2 Materials and methods

### 2.1 Patients

We consecutively recruited a retrospective cohort of patients who were hospitalized and treated at the First Affiliated Hospital of Wenzhou Medical University between September 2012 and December 2020 with severe liver injury (TB ≥ 5 mg/dl, INR ≥ 1.5, or prothrombin activity <40% according to APASL criteria 2019) from Chronic hepatitis B (CHB) (Sarin et al., 2019). CHB patients were enrolled based on the 2009 AASLD guidelines (Lok and McMahon, 2009). Patients' outcomes were organ failures and 28-day, 3-month, and 12-month survival. The exclusion criteria were as follows: (i) liver disease etiology: viral hepatitis other than HBV, alcoholic liver disease, drug-induced liver injury, autoimmune hepatitis, etc.; (ii) acute liver failure; (iii) patients with malignant diseases; (iv) known recent myocardial infarction (<6 months) or stroke with residual defects; (v) severe non-liver-related reasons for admission; (vi) patients with either respiratory failure, circulation failure, or renal failure and a life expectancy <48 h; (vii) patients with liver transplantation; and (viii) patients who lost follow-up or had deficient data. This study conformed to the principles of the Declaration of Helsinki and was approved by the Institute Ethics Committee (KY2021-R055). Informed consent was waived due to the observational nature of the study.

### 2.2 Data collection

Using data from the hospital information system and medical documents, we obtained information regarding age, gender, diabetes, hypertension, nucleoside analog discontinuance, laboratory measurements (e.g., white blood cell counts, platelets, bilirubin, serum albumin, alanine aminotransferase, aspartate aminotransferase, INR, creatinine levels, and sodium), complication events (ascites, HE, upper GI bleeding, and infection events), and events of organ failure. Hepatic encephalopathy was diagnosed according to West Haven criteria (Vilstrup et al., 2014). HE grade 2/3/4 was considered overt HE. Patients with ACLF were routinely administered lactulose to achieve two loose stools per day for treatment or prevention of hepatic encephalopathy development, according to guideline practice (Vilstrup et al., 2014). Infection events were diagnosed by combining microbial detection with clinical or laboratory signs. Prognostic scores, including Child-Pugh, the Model for End-Stage Liver Disease (MELD), MELD-sodium (MELD-NA), the Chronic Liver Failure Consortium Organ Failure score (CLIF-SOFA), the CLIF-Consortium-ACLF (CLIF-C ACLF), and the APASL ACLF Research Consortium (AARC) score, were calculated using parameters obtained at baseline. The 28-day, 3-month, and 12-month mortality was determined by telephone follow-up.

### 2.3 Measurement of ammonia

Ammonia was measured using standard operating protocols with a high degree of accuracy. Venous samples were collected in cooled EDTA tubes and rapidly transported on ice to the laboratory for spectrophotometric assay. All ammonia levels and the time-point of measurements of each patient during hospitalization were recorded. Baseline ammonia was defined as an ammonia level measured on admission. The highest and lowest ammonia levels during hospitalization were considered as Peak ammonia and Trough ammonia. For standardizing ammonia levels across different laboratories, we converted the ammonia measurement to a ratio of AMM-ULN [serum ammonia (μmol/L)/reference laboratory upper limit of normal for ammonia (60 μmol/L)] to correct for sample collection bias (Tranah et al., 2022). The mean ammonia levels, which were measured during days 0–3 and days 4–7 after admission, were calculated and then compared by subtraction. Changes in ammonia were subsequently recorded.

### 2.4 Statistical analyses

Continuous data were expressed as mean standard deviation or median (25^th^ to 75^th^ percentiles) as appropriate. Categorical data were presented as proportions. A comparison between continuous variables was performed using the Student's *t-*test or the Mann-Whitney U test. A chi-square or Fisher's exact test was used for the comparison of categorical variables. The receiver operating characteristic (ROC) curve and the area under the curve (AUC) were conducted for the assessment of accuracy in predicting the outcome for AMM-ULN (Baseline, Peak, Trough). The optimal cut-off values for AMM-ULN (Baseline, Peak, Trough) were determined by X-tile software (Version 3.6.1, Yale University, New Haven, CT, United States). The De Long method was used for the area under ROC (AUROC) comparisons. Univariate analysis and a multivariate Cox proportional hazards model were performed to identify significant predictors for overt HE and 28-day mortality. Survival curves were calculated using the Kaplan-Meier method and compared with the log-rank test. The significance level was set at P < 0.05. SPSS V.20.0, MedCalc V.14.8.1, and Origin 2019b software were used for the analyses.

## 3 Results

### 3.1 Baseline characteristics

A total of 276 patients with HBV-ACLF were recruited. Patients were mostly male (85.8%), with a median age of 42 (34–53) years. Nearly one-fifth of patients discontinued nucleotide analog therapy. The frequency of organ failures developed during hospitalization is as follows: respiratory failure 21 (7.6%), circulation failure 24 (8.7%), and renal failure 14 (5.1%). Complication events including HE, ascites, gastrointestinal hemorrhage, and bacterial infections were present in 65 (23.6%), 161 (58.7%), 21 (7.6%), and 140 (50.7%) patients, respectively. In 28-day mortality, 3-month mortality, and 1-year mortality were 19.2, 25.7, and 28.2%, respectively. Seven patients were documented with a history of prior episodes of HE. Among them, three patients underwent more than one prior episode of HE. A description of the demographics and clinical characteristics of HE in various grades can be found in Table 1.

**Table 1:** Comparison of patients' characteristics stratified by different grades of HE (*n* = 276).

**Baseline characteristics**	**Grade 0/1 HE (*N* = 225)**	**Grade 2/3/4 HE (*N* = 51)**	**P**
Age (years)	43.2 (±12.9)	48.3 (±12.8)	**0.012**
Gender (male)	193 (85.7%)	44 (86.3%)	0.927
Diabetes mellitus	25 (11.1%)	12 (23.5%)	**0.019**
Hypertension	28 (12.4%)	9 (17.6%)	0.325
Drug discontinuance	47 (20.8%)	9 (17.6%)	0.603
Alcohol	33 (14.6%)	12 (23.5%)	0.122
Cirrhosis	79 (35.1%)	28 (54.9%)	**0.009**
**Laboratory values**			
WBC	6.3 (4.9–8.2)	8.2 (5.1–11.2)	**0.022**
Platelets	119.0 (86.0–157.0)	94.0 (60.0–147.0)	0.177
Bilirubin (mg/dl)	12.7 (7.2–18.8)	15.8 (11.2–21.1)	**0.027**
Albumin (g/dl)	30.6 (±5.2)	29.7 (±5.7)	0.281
ALT	505.5 (157.5–1,114.0)	411.0 (144.0–1,056.0)	0.490
AST	347.5 (144.5–803.5)	308.0 (176.0–872.0)	0.707
INR	2.1 (1.8–2.3)	2.9 (2.1–3.6)	**<0.001**
PTA	38.0 (32.0–45.0)	26.0 (21.0–36.0)	**<0.001**
Creatinine (mg/dl)	0.7 (0.6–0.8)	0.7 (0.6–0.9)	0.148
Sodium (mmol/L)	137 (135–139)	136 (133–138)	**0.033**
Lactate (mmol/L)	2.7 (2.1–3.3)	3.0 (2.5–5.4)	**0.029**
AFP	73.3 (28.0–192.7)	41.2 (13.9–89.5)	**0.007**
Baseline ammonia (mmol/L)	52.5 (36.0–72.0)	70.0 (43.0–100.0)	**0.003**
AMM-ULN (Baseline)	0.85 (0.53–1.19)	1.16 (0.71–1.67)	**0.001**
Peak ammonia (mmol/L)	80.0 (59.5–106.0)	152.0 (114.0–191.0)	**<0.001**
AMM-ULN (Peak)	1.30 (0.86–1.73)	2.53 (1.90–3.18)	**<0.001**
Trough ammonia (mmol/L)	38.0 (27.0–53.0)	54.0 (33.0–79.0)	**<0.001**
AMM-ULN (Trough)	0.61 (0.40–0.87)	0.90 (0.55–1.32)	**<0.001**
Bacterial infections	101 (44.8%)	39 (76.5%)	**<0.001**
Upper GI bleeding	5 (2.2%)	16 (31.3%)	**<0.001**
Ascites	125 (55.5%)0	36 (70.6%)	0.053
Respiratory failure	3 (1.3%)	18 (35.3%)	**<0.001**
Circulation failure	7 (3.1%)	17 (33.3%)	**<0.001**
Kidney failure	4 (1.7%)	10 (19.6%)	**<0.001**
**Scores**			
Child-Pugh	10 (10–12)	13 (12–14)	**<0.001**
MELD	21.0 (18.4–23.6)	26.3 (22.0–29.9)	**<0.001**
MELD-NA	22.6 (19.3–25.3)	27.3 (23.8–31.2)	**<0.001**
CLIF-SOFA	7 (6–7)	11 (10–12)	**<0.001**
CLIF-C ACLF	28.7 (23.7–34.0)	45.2 (39.1–56.0)	**<0.001**
AARC	7 (6–9)	11 (9–12)	**<0.001**
**ACLF**			
Grade I	115 (51.1%)	2 (3.9%)	**<0.001**
Grade II	94 (41.7%)	21 (41.2%)	
Grade III	16 (7.1%)	28 (54.9%)	
28-day mortality (%)	15 (6.7%)	38 (74.5%)	**<0.001**
3-month mortality (%)	25 (11.1%)	46 (90.2%)	**<0.001**
1-year mortality (%)	31 (13.7%)	47 (92.2%)	**<0.001**

Bold values represent statistical significance. WBC, white blood cell counts; ALT, alanine aminotransferase; AST, aspartate aminotransferase; INR, international normalized ratio; PTA, prothrombin activity; AFP, alpha-fetoprotein; AMM-ULN, ammonia level corrected to the upper limit of normal; GI, gastrointestinal; ACLF, acute-on-chronic liver failure.

### 3.2 Factors associated with HE

A total of 51 participants (18.4%) were noted to have overt HE. Peak AMM-ULN was significantly higher in patients with HE grade 2/3/4 compared to those with HE grade 0/1 (2.53 [1.90–3.18] vs. 1.30 [0.86–1.73], *P* < 0.001) (Supplementary Figure S1A). Baseline AMM-ULN and Trough AMM-ULN presented similar results (1.16 [0.71–1.67] vs. 0.85 [0.53–1.19], *P* < 0.001; 0.90 [0.55–1.32] vs. 0.61 [0.40–0.87], *P* < 0.001). On univariate analysis, laboratory values including white blood cell counts (WBC), platelets (PLT), bilirubin, INR, PTA, sodium, lactate, alpha-fetoprotein (AFP), and AMM-ULN (Baseline, Peak, Trough) were predictive of HE grade 2/3/4. To analyze which factors remained associated with HE grade 2/3/4, different multivariate models were constructed. In a multivariate model including all significant continuous variables but excluding organ failures, age (OR, 1.041; *P* = 0.040), INR (OR 1.258; *P* = 0.006), and Peak AMM-ULN (OR 1.028; *P* < 0.001) remained associated with overt HE. In a multivariate analysis including complication events and organ failures, Peak AMM-ULN (OR 1.031; *P* < 0.001), gastrointestinal hemorrhage (OR, 9.015; *P* < 0.010), and respiratory failures (OR, 23.707; *P* = 0.001) remained independently associated with overt HE (Supplementary Table S1).

Those with overt HE had much more severe clinical conditions, as indicated by the significantly higher proportion of complication event development and higher Child-Pugh, MELD, MELD-Na, CLIF-SOFA, CLIF-C ACLF, and AARC scores. Patients with overt HE had remarkably increased mortality at 28 days, 3 months, and 1 year compared to mortality in those with grade 0/1 HE (74.5 vs. 6.7%, 90.2 vs. 11.1%, 92.2 vs. 13.7%, respectively; *P* < 0.001) (Table 1).

**Figure 1:**
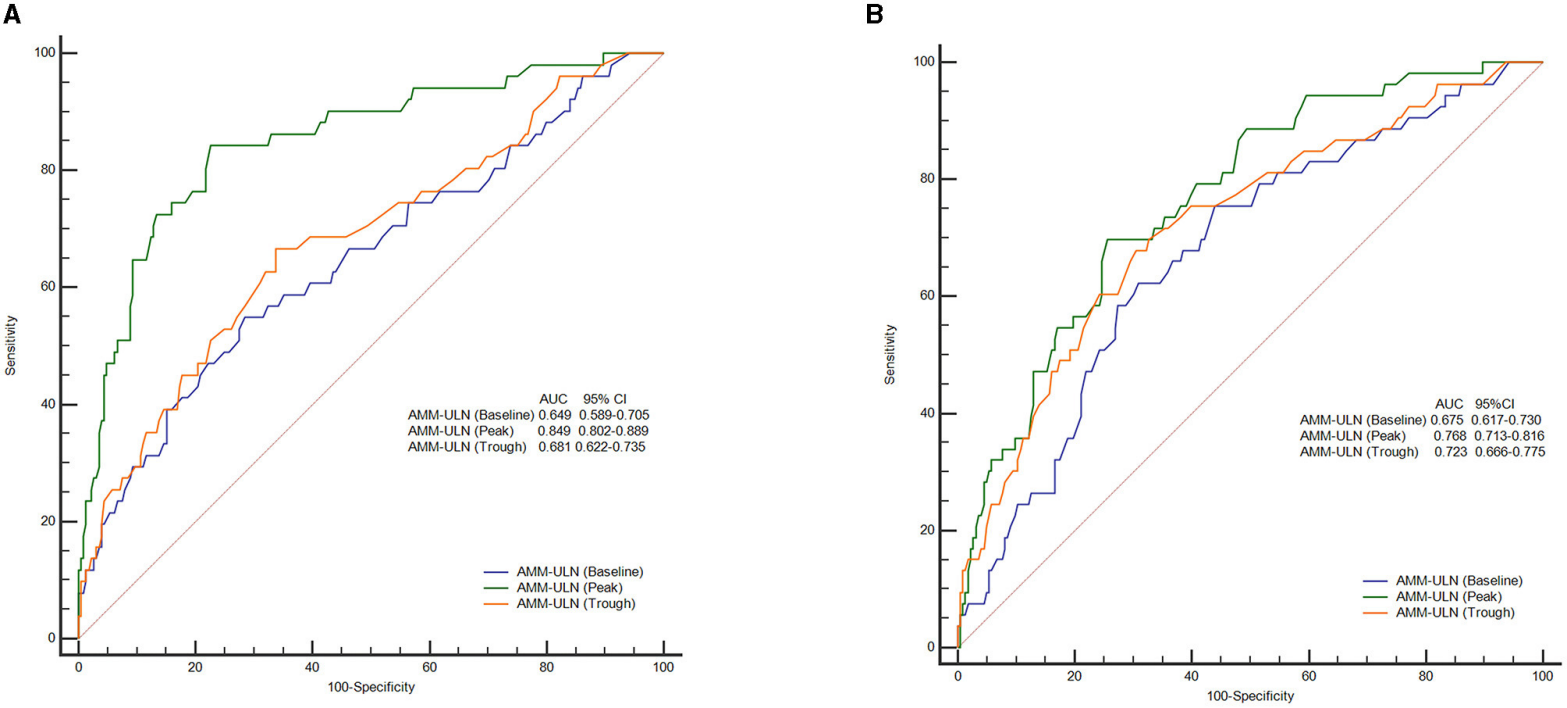
ROC curves of AMM-ULN (Baseline, Peak, Trough) in predicting overt HE **(A)** and 28-day mortality **(B)**. Peak AMM-ULN demonstrates improved predictive performance (84.9%) for the overt HE events when compared against Baseline and Trough AMM-ULN (64.9 and 68.1%, *P* < 0.001). For prediction of 28-day mortality, Peak AMM-ULN was significantly better than Baseline AMM-ULN (76.8 vs. 67.5%, *P* = 0.025), and showed a tendency to perform better than Trough AMM-ULN (76.8 vs. 72.3%, *P* = 0.294). ROC, receiver operating characteristics curve analysis; AMM-ULN, ammonia level corrected to the upper limit of normal; HE, hepatic encephalopathy.

The values of AMM-ULN (Baseline, Peak, Trough) for predicting overt HE were evaluated by AUROC. The AUROC for Peak AMM-ULN was 84.9% for overt HE, significantly higher than the Baseline AMM-ULN and Trough AMM-ULN (64.9%, *P* < 0.001; 68.1%, *P* < 0.001), as shown in Figure 1A. The optimal cut-off of Peak AMM-ULN as a predictor of overt HE was 1.7, with a sensitivity of 84.3% and a specificity of 77.3%.

### 3.3 AMM-ULN associated with mortality

The 28-day mortality of the included patients was 19.2% (*n* = 53). Non-survivors were older (42.7 vs. 50.3, *P* < 0.001) and more likely to have alcohol abuse (*P* = 0.002) and cirrhosis (*P* = 0.019). Peak AMM-ULN was significantly higher in non-survivors compared with survivors (2.13 [1.51–3.00] vs. 1.30 [0.85–1.80], *P* < 0.001) (Supplementary Figure S1B). Non-survivors had a higher percentage of complication events and organ failures (*P* ≤ 0.001). It was found that non-survivors had higher Child-Pugh, MELD, MELD-NA, CLIF-SOFA, CLIF-C ACLF, and AARC scores at baseline compared with survivors (13 vs. 11, 28.7 vs. 21.0, 30.3 vs. 22.2, 10 vs. 7, 45.5 vs. 28.6, 11 vs. 7, respectively; *P* < 0.001) (Table 2).

**Table 2:** Comparison of characteristics of patients with HBV-ACLF who survived and those who died (*n* = 276).

**Baseline characteristics**	**Survived (*N* = 223)**	**Died (*N* = 53)**	**P**
Age (years)	42.7 (±12.4)	50.3 (±13.8)	**<0.001**
Gender (male)	192 (86.1%)	45 (84.9%)	0.823
Diabetes mellitus	26 (11.7%)	11 (20.8%)	**0.081**
Hypertension	26 (11.7%)	11 (20.8%)	0.081
Drug discontinuance	48 (20.8%)	8 (17.8%)	0.647
Alcohol	29 (13.0%)	16 (30.2%)	**0.002**
Cirrhosis	79 (35.4%)	28 (52.8%)	**0.019**
**Laboratory values**			
WBC	6.2 (4.8–8.2)	8.5 (6.0–12.3)	**<0.001**
Platelets	119 (85.5–157.5)	97 (58–142)	0.072
Bilirubin (mg/dl)	12.6 (7.1–17.3)	20.4 (11.9–25.5)	**<0.001**
Albumin (g/dl)	30.7 (±5.2)	29.1 (±5.6)	0.042
ALT	530.0 (169.2–1,152.5)	313.0 (121.0–855.5)	0.115
AST	372.5 (150.7–828.3)	264.0 (143.5–810.0)	0.240
INR	2.1 (1.8–2.3)	3.0 (2.5–3.9)	**<0.001**
PTA	39.0 (34.0–44.3)	25.0 (20.5–30.5)	**<0.001**
Creatinine (mg/dl)	0.7 (0.6–0.8)	0.8 (0.6–1.3)	**0.001**
Sodium (mmol/L)	137 (135–139)	136 (131–138)	**<0.001**
Lactate (mmol/L)	2.7 (2.1–3.2)	2.9 (2.4–4.9)	**0.029**
AFP	73.3 (28.8–186.2)	40.2 (12.8–93.6)	**0.002**
Baseline ammonia (mmol/L)	52.0 (35.0–72.3)	72.0 (51.5–93.5)	**<0.001**
AMM-ULN (Baseline)	0.83 (0.51–1.18)	1.20 (0.85–1.56)	**<0.001**
Peak ammonia (mmol/L)	81.0 (59.0–113.5)	128.0 (91.0–180.5)	**<0.001**
AMM-ULN (Peak)	1.30 (0.85–1.80)	2.13 (1.51–3.00)	**<0.001**
Trough ammonia (mmol/L)	38.0 (26.0–52.5)	56.0 (40.0–77.0)	**<0.001**
AMM-ULN (Trough)	0.61 (0.40–0.85)	0.93 (0.67–1.28)	**<0.001**
Bacterial infections	98 (43.9%)	42 (79.2%)	**<0.001**
Upper GI bleeding	6 (2.7%)	15 (28.3%)	**<0.001**
Ascites	119 (53.6%)	42 (79.2%)	0.001
HE (grade 3/4)	9 (4.0%)	36 (67.9%)	**<0.001**
Respiratory failure	6 (2.7%)	15 (28.8%)	**<0.001**
Circulation failure	7 (3.1%)	17 (32.7%)	**<0.001**
Kidney failure	4 (1.8%)	10 (18.9%)	**<0.001**
**Scores**			
Child-Pugh	11 (10–12)	13 (11–14)	**<0.001**
MELD	21.0 (18.2–23.1)	28.7 (23.9–32.9)	**<0.001**
MELD-NA	22.2 (19.3–24.9)	30.3 (26.3–34.6)	**<0.001**
CLIF-SOFA	7 (6–7)	10 (9–12)	**<0.001**
CLIF-C ACLF	28.6 (23.6–33.7)	45.5 (39.9–58.1)	**<0.001**
AARC	7 (6–9)	11 (10–12.5)	**<0.001**
**ACLF**			
Grade I	115 (51.6%)	2 (3.8%)	**<0.001**
Grade II	95 (42.6%)	20 (37.7%)	
Grade III	13 (5.8%)	31 (58.5%)	

Bold values represent statistical significance. WBC, white blood cell counts; ALT, alanine aminotransferase; AST, aspartate aminotransferase; INR, international normalized ratio; PTA, prothrombin activity; AFP, alpha-fetoprotein; AMM-ULN, ammonia level corrected to the upper limit of normal; HE, hepatic encephalopathy; GI, gastrointestinal; ACLF, acute-on-chronic liver failure.

In the univariate analysis, aging, cirrhosis, WBC, bilirubin, INR, PTA, creatinine, sodium, lactate, AMM-ULN (Baseline, Peak, Trough), bacterial infection, gastrointestinal hemorrhage, ascites, overt HE, and organ failures were found to be related to 28-day mortality. Multivariate analysis showed that age, bilirubin, INR, creatinine, Peak AMM-ULN, gastrointestinal hemorrhage, overt HE, and circulation failure were independently associated with 28-day mortality (Table 3). These results indicated that Peak AMM-ULN was an independent factor associated with 28-day mortality. Moreover, Peak AMM-ULN was demonstrated to be positively correlated to various prognostic scores, including Child-Pugh (r = 0.316, *P* < 0.001), MELD (r = 0.193, *P* = 0.001), MELD-NA (r = 0.173, *P* = 0.004), CLIF-SOFA (r = 0.460, *P* < 0.001), CLIF-ACLF (r = 0.371, *P* < 0.001), and AARC (r = 0.373, *P* < 0.001), as shown in Supplementary Figure S2. Notably, we compared the values of AMM-ULN (Baseline, Peak, Trough) of patients with different grades of ACLF. As shown in Supplementary Figure S3, AMM-ULN (Baseline, Peak, Trough) levels of patients with ACLF grade 3 were significantly higher than those of patients with ACLF grade 1 (*P* < 0.001, *P* < 0.001, and *P* = 0.001, respectively). Moreover, our data also demonstrated that patients with a higher AMM-ULN (Baseline, Peak, Trough) have a much higher proportion of patients in Grade 3 compared to their counterparts (Supplementary Figure S4). Thus, these results indicated that high ammonia levels were positively correlated with the severity of ACLF.

**Table 3:** Univariate and multivariate analysis for predictors of 28-day mortality.

**Baseline characteristic**	**Univariate model**		**Multivariate model**	
	**HR**	**P**	**HR**	**P**
Age (years)	**1.037 (1.017–1.058)**	**<0.001**	**1.029 (1.001–1.059)**	**0.046**
Gender (male/female)	0.908 (0.428**–**1.925)	0.800		
Diabetes mellitus	1.806 (0.930**–**3.509)	0.081		
Hypertension	1.808 (0.931**–**3.511)	0.081		
Drug discontinuance	0.578 (0.261**–**1.280)	0.276		
Alcohol	2.405 (1.337**–**4.325)	0.003		
Cirrhosis	**1.852 (1.080–3.177)**	**0.025**		
**Laboratory values**				
WBC	**1.133 (1.086–1.183)**	**<0.001**		
Platelets	0.995 (0.989**–**1.000)	0.068		
Bilirubin (mg/dl)	**1.083 (1.052–1.115)**	**<0.001**	**1.093 (1.044–1.144)**	**<0.001**
Albumin (g/dl)	0.951 (0.905**–**1.000)	0.048		
ALT	1.000 (0.999**–**1.000)	0.130		
AST	1.000 (0.999**–**1.000)	0.264		
INR	**2.594 (2.149–3.130)**	**<0.001**	**2.214 (1.471–3.334)**	**<0.001**
PTA	**0.881 (0.854–0.910)**	**<0.001**		
Creatinine (mg/dl)	**2.646 (1.946–3.598)**	**<0.001**	**1.864 (1.153–3.015)**	**0.011**
Sodium (mmol/L)	**0.886 (0.840–0.936)**	**<0.001**		
Lactate (mmol/L)	**1.213 (1.098–1.340)**	**<0.001**		
AFP	0.998 (0.995**–**1.000)	0.057		
AMM-ULN (Baseline)	**1.012 (1.005–1.018)**	**<0.001**		
AMM-ULN (Peak)	**1.007 (1.005–1.009)**	**<0.001**	**1.026 (1.013–1.039)**	**<0.001**
AMM-ULN (Trough)	**1.025 (1.016–1.034)**	**<0.001**		
Bacterial infections	**4.248 (2.186–8.255)**	**<0.001**		
Upper GI bleeding	**7.829 (4.282–14.313)**	**<0.001**	**4.164 (1.685–10.292)**	**0.002**
Ascites	**1.504 (1.504–5.675)**	**0.002**		
HE (grade 3/4)	**20.137 (10.963–36.989)**	**<0.001**	**9.716 (4.395–21.478)**	**<0.001**
Respiratory failure	**7.978 (4.350–14.630)**	**<0.001**		
Circulation failure	**7.441 (4.143–13.365)**	**<0.001**	**0.038(0.146–0.986)**	**0.047**
Kidney failure	**6.316 (3.164–12.607)**	**<0.001**		

Bold values represent statistical significance. WBC, white blood cell counts; ALT, alanine aminotransferase; AST, aspartate aminotransferase; INR, international normalized ratio; PTA, prothrombin activity; AFP, alpha-fetoprotein; AMM-ULN, ammonia level corrected to the upper limit of normal; GI, gastrointestinal.

The prognostic values of AMM-ULN (Baseline, Peak, Trough) for predicting outcomes were assessed by analyzing the AUROC. AUROC of Peak AMM-ULN was 76.8% for 28-day mortality, higher than Baseline AMM-ULN (65.5%, *P* = 0.025) but not significantly higher than Trough AMM-ULN (72.3%, *P* = 0.294) (Figure 1B). The sensitivity and specificity were 69.8 and 74.4% for the Peak AMM-ULN level.

**Figure 2:**
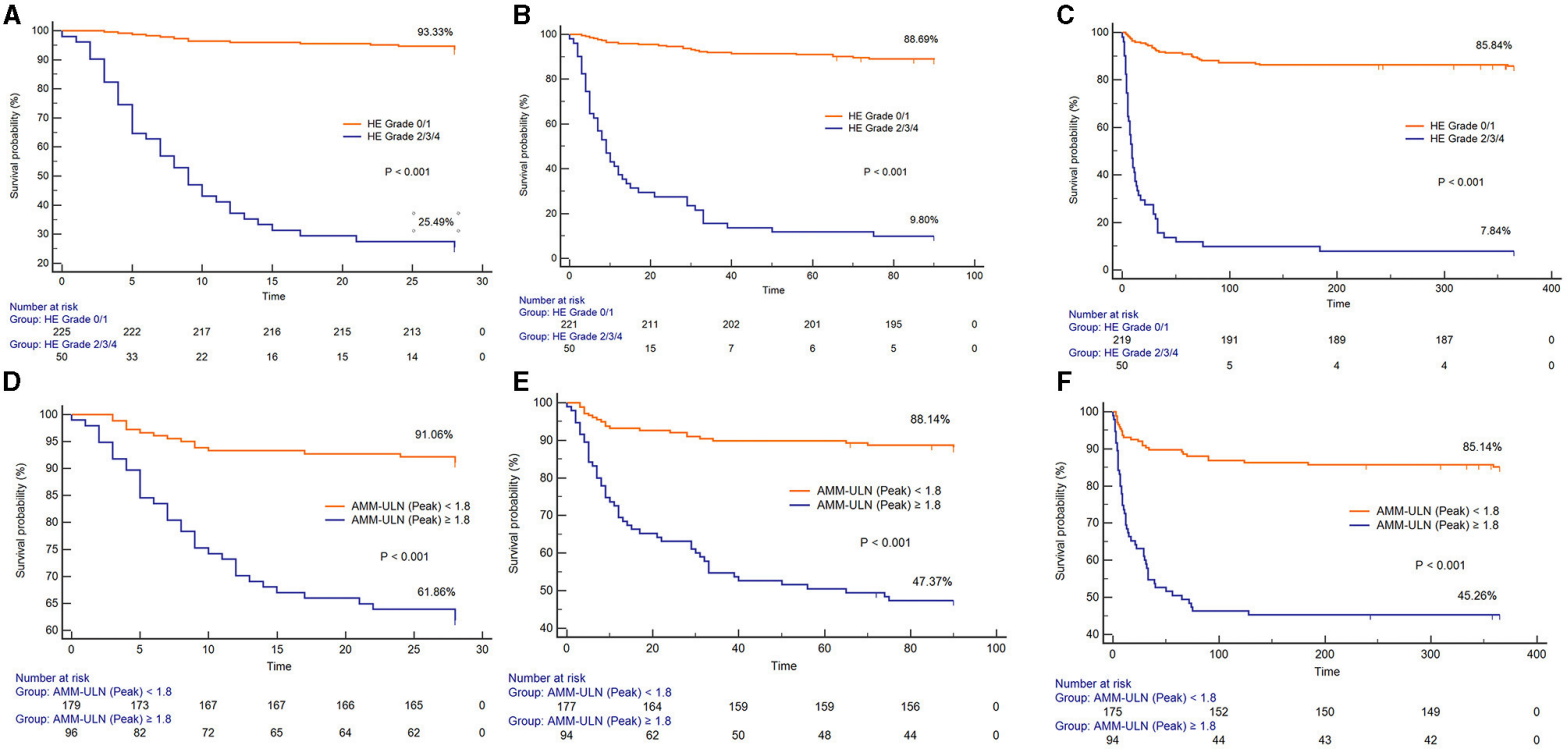
Kaplan-Meier graph of 28-day survival, 3-month survival, and 1-year survival stratified by HE grades 0–1 or grades 2–4. **(A–C)** Kaplan-Meier graph of 28-day survival, 3-month survival, and 1-year survival stratified by Peak AMM-ULN <1.8 or ≥1.8 **(D–F)**. The mortality among patients with HE grade 2/3/4 and Peak AMM-ULN ≥1.8 were significantly higher than those with HE grade 0/1 and Peak AMM-ULN <1.8 (log-rank test, *P* < 0.001). HE, hepatic encephalopathy; AMM-ULN, ammonia level corrected to the upper limit of normal.

X-tile software was subsequently used to determine the optimal cut-off values of AMM-ULN (Baseline, Peak, Trough) for 28-day mortality, which were 1.1, 1.8, and 0.9, respectively (Supplementary material). The Kaplan-Meier survival curves of patients stratified by HE grades 0–1 or grades 2–4 and Peak AMM-ULN ≥1.8 or <1.8 for 28-day, 3-month, and 1-year mortality are shown in Figure 2. Patients with overt HE and Peak AMM-ULN ≥1.8 had significantly higher mortality than patients with no/mild HE and Peak AMM-ULN <1.8 (log-rank test, *P* < 0.001). Similar results were observed for Baseline AMM-ULN and Trough AMM-ULN (Supplementary Figure S5). As a result of these findings, patients with overt HE or AMM-ULN ≥1.8 have significantly higher mortality compared to patients with no/mild HE or AMM-ULN <1.8.

To explain the impact of bacterial infection on ammonia levels, we further analyzed the effect of serum ammonia on patients' 28-day mortality according to the presence or absence of bacterial infection. As shown in Supplementary Figure S6, regardless of the presence or absence of infection, patients with AMM-ULN ≥1.8 have significantly higher mortality compared to patients with AMM-ULN <1.8.

### 3.4 HE development and mortality among patients with Peak AMM-ULN appearing at different time points after admission

**Figure 3:**
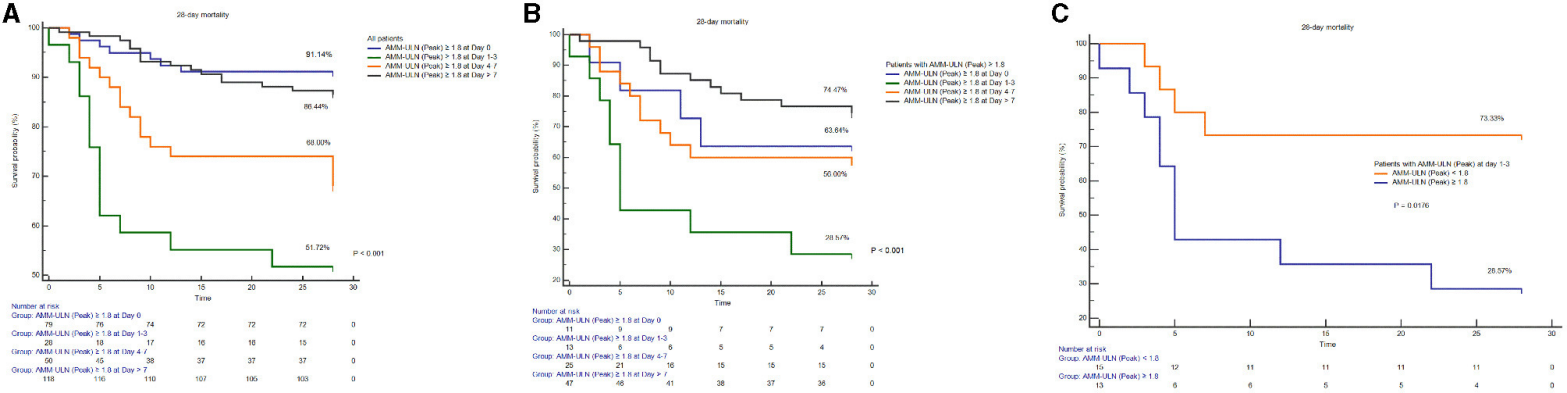
Comparison of survival probability among patients with Peak AMM-ULN appearing at different time points (day 0, days 1–3, days 4–7, day >7) after admission. Among all patients **(A)** or patients with Peak AMM-ULN ≥1.8 **(B)**, patients of group (days 1–3) both have significantly lower survival probability compared to the patients of other time points groups (log-rank test, *P* < 0.001). Among patients of group (days 1–3), patients with Peak AMM-ULN ≥1.8 conferred lower survival than those with Peak AMM-ULN <1.8 **(C)**. AMM-ULN, ammonia level corrected to the upper limit of normal.

Patients were categorized into four groups based on the time points (day 0, days 1–3, days 4–7, days >7) of Peak AMM-ULN appearance after admission. The values of Peak AMM-ULN were significantly higher in patients with HE 2/3/4 and survivors than those in patients with HE 0/1 and non-survivors in each group (Supplementary Figure S7). Patients in the group (Days 1–3) had a higher proportion of overt HE development compared to patients in other time points, although the difference is insignificant due to the limited sample (Supplementary Figures S8A, B). Among patients in groups (Days 1–3), patients with Peak AMM-ULN≥1.8 had a 64% proportion of overt HE development, while it was 0% in patients with Peak AMM-ULN <1.8 (*P* < 0.001) (Supplementary Figure S8C). In addition, patients with Peak AMM-ULN appearing on days 1–3 had a lower survival probability compared to patients from other time points in the whole series of patients. When the comparison was restricted to patients with Peak AMM-ULN ≥1.8, patients in groups (days 1–3) showed significantly lower survival probabilities as well (Figure 3). The above results show that HBV-ACLF patients with Peak AMM-ULN during the first 3 days of hospitalization develop a higher proportion of HE and have higher 28-day mortality.

### 3.5 Elevation in ammonia levels indicated a poor clinical outcome

We calculated the mean ammonia levels, which were measured during days 0–3 and days 4–7 after admission. The change in ammonia level was computed and then classified patients into the ammonia-increased group (*n* = 115) or the ammonia-decreased group (*n* = 161). Patients with increased ammonia, compared to those with decreased ammonia, developed a higher proportion of severe complications, including severe HE (27.8 vs. 8.1%, *P* < 0.001), bacterial infections (63.4 vs. 41.6%, *P* < 0.001), gastrointestinal hemorrhage (12.2 vs. 4.3%, P = 0.015) moderate-severe ascites (33.9 vs. 20.9%, P = 0.001). Additionally, the prognostic scores, including Child-Pugh, MELD, MELD-NA, CLIF-SOFA, CLIF-C ACLF, and AARC, were significantly higher in patients with increased ammonia than those with decreased ammonia (12 [10–13] vs. 10 [10–12], *P* < 0.001; 23 [18–27] vs. 21[18–23], *P* = 0.01; 25 [18–27] vs. 22 [19–24], *P* < 0.001; 7 [6–10] vs. 7 [6–8], *P* < 0.001; 34.9 [27.7–44.6] vs. 28.4 [23.4–34.0], *P* < 0.001; 9 [7–11] vs. 7 [6–9], *P* < 0.001). The proportion of ACLF Grade III is markedly higher in the ammonia-increased group than in ammonia-decreased group as well (27.9 vs. 7.4%, *P* < 0.001). Worsening of ammonia was associated with a 28-day mortality of 29.6% (*n* = 34) compared to 11.8% (*n* = 19) in those with decreased ammonia (*P* < 0.001). Similar results were also presented for 3-month and 1-year mortality (Supplementary Table S2).

In addition, we further analyzed the difference in clinical outcomes between groups of patients in which the ammonia levels decreased after the peak value had been obtained. As data showed in Supplementary Table S3, the proportion of HE episodes was significantly lower in a group of patients with Peak AMM-ULN decrease than their counterparts (19.2 vs. 30.8%, *P* = 0.030), and a higher rate of reduction in HE grades was observed in these patients (37.2 vs. 10.3%, *P* = 0.014). Furthermore, the 28-day, 3-month, and 1-year mortalities of patients whose ammonia levels decreased after the Peak ammonia obtained were significantly lower compared with their counterparts (14.8 vs. 38.2%, *P* = 0.01; 18.5 vs. 40.4%, *P* < 0.001; 21.0 vs. 43.6%, *P* < 0.001), as presented in Supplementary Figure S9.

## 4 Discussion

This study explores the predictive role of the dynamics of ammonia levels in a population of HBV-ACLF patients. This cohort included a highly homogeneous population that was admitted to the general ward due to a severe flare-up of chronic HBV. We found that ammonia levels were a statistically significant risk for overt HE and mortality in patients with HBV-ACL. Peak AMM-ULN ≥ 1.8 was independently associated with overt HE and mortality. Peak AMM-ULN appearing on days 1–3 after admission and elevation of ammonia within 7 days during hospitalization suggested a poor prognosis.

ACLF is an acute deterioration of chronic liver disease characterized by high short-term mortality, which requires accurate initial clinical decision-making. HE is a common complication of decompensated cirrhosis and ACLF and manifests in a range of ways, from mild cognitive alteration to coma. HE development in hospitalized patients with ACLF has been proven to be associated with high mortality (Cordoba et al., 2014; Romero-Gomez et al., 2015). Consistently, our data showed that overt HE correlates with a markedly higher risk of 28-day mortality (74.5 vs. 6.7%, *P* < 0.001). Sawhney et al. (2016) demonstrated that mortality in ACLF patients with HE was higher than that without HE (66% vs. 33%), regardless of ACLF severity. Moreover, they also found mortality was increased in patients with higher HE grades, which is consistent with our study (Sawhney et al., 2016). Recently, another study that included ACLF patients of all etiologies (55.0% of alcohol) performed by the APASL ACLF Working Party first described an association between HEs dynamic evolution and survival. Similar to our study, their data indicated that ACLF patients with a progressive course or HE grades III–IV had remarkably worse outcomes (Verma et al., 2021). For the first time, we explored the clinical utility of ammonia variation in a highly homogeneous population that was predominately admitted to the general ward due to a severe flare-up of chronic HBV.

Serum ammonia levels are considered to play a major role in the pathophysiology of HE in liver diseases. The mechanism explaining hyperammonemia in HE is classically referred to as direct neurotoxicity, which leads to astrocyte swelling, neuroinflammation, cell signaling, and neurotransmission impairment. In recent years, increasing studies have reported that ammonia toxicity also affects other organs, including the liver and muscles, and contributes to immune dysfunction, sarcopenia, and portal hypertension (Shawcross et al., 2008; Jalan et al., 2016; Nardelli et al., 2019; Deutsch-Link et al., 2022). Clinical practitioners have been struggling with how to incorporate serum ammonia levels into clinical practice. Unfortunately, although serum ammonia is broadly accepted to be associated with HE, neither a recognized diagnostic threshold nor a standard upper limit of normal is proposed. Jalan et al. (2016) demonstrated that the optimal cut-off of baseline ammonia for overt HE was 79.5 μmol/L in cirrhotic patients. Those patients with an ammonia level of ≥79.5 μmol/L had significantly higher mortality (Shalimar et al., 2019). However, ammonia levels vary in clinical practice and there is an overlap between patients who have different grades of HE. Another challenge to the widespread usage of ammonia is the non-standardized protocols of measurement in different laboratories and the reflected differences in the range and distribution of values (Howanitz et al., 1984; Bajaj et al., 2020). Recently, a landmark study performed by Tranah et al. (2022) transformed the ammonia level to a ratio of the patient's ammonia level using the formula: AMM-ULN = serum ammonia (μmol/L)/reference laboratory upper limit of normal for ammonia (μmol/L) to avoid bias. They found that AMM-ULN > 1.4 defines the risk of hospitalization due to liver-related complications and mortality in clinically stable outpatients with cirrhosis (Tranah et al., 2022). To support the proposal of using AMM-ULN to harmonize ammonia levels, we converted the ammonia level to AMM-ULN as well. We determined that Peak AMM-ULN ≥1.8 correlated with a significantly higher frequency of overt HE and mortality. Consistent with Shalimar et al.'s (2020) research that persistent hyperammonemia during the first 3 days of hospitalization indicates a worse outcome, our patients with Peak AMM-ULN appearing at days 1–3 after admission had a higher frequency of overt HE development and mortality, which indicates that the dynamic change of ammonia levels within 3 days is critical for the prognosis of ACLF patients.

In the past decades, the prognostic role of serum ammonia in patients with chronic liver diseases has been under debate. In recent years, emerging studies have suggested that hyperammonemia predicts poor outcomes in patients with ACLF (Hu et al., 2020; Shalimar et al., 2020; Zhang et al., 2020; Chiriac et al., 2021). In clinical practice, drugs including lactulose, rifaximin, and L-ornithine-L-aspartate can lower ammonia levels by either decreasing ammonia production or improving ammonia clearance. However, the use of ammonia as a biomarker to guide therapy in chronic liver diseases was limited in clinical trials because of a lack of standardization of sample measurement protocol, inadequate timing of ammonia assessment, and inconstant ammonia assessment (Sharma et al., 2017; Rahimi et al., 2021; Rose et al., 2021). Interestingly, a recent double-blind random clinical trial showed a significantly faster clinical improvement of symptoms in ornithine phenylacetate-treated patients with initially confirmed baseline hyperammonemia (Rahimi et al., 2021). Their results implicate that patients with hyperammonemia were more likely to respond to the therapy. Thus, an assessment of ammonia levels may help differentiate treatment responders from non-responders. Further studies and more data are warranted to fully support the approach of using ammonia to monitor and adapt treatments in clinical practice.

To date, studies directly analyzing the prognostic effect of dynamic ammonia in populations with HBV-ACLF are scarce. Research performed by Hu et al. has determined that baseline hyperammonemia is a strong indicator of the prognosis of patients with ACLF according to APASL criteria, in which they included 106 patients with HBV-ACLF (Hu et al., 2020). Another study that enrolled 127 patients with HBV-ACLF according to the Chinese Group on the Study of Severe Hepatitis B (COSSH)-ACLF criteria also showed similar results (Zhang et al., 2020). Differing from the above studies, we further describe the clinical value of the dynamics of ammonia estimation in patients with HBV-ACLF. Remarkably, by comparing the MELD score and mortality, their patients were more severe than ours. This is explained by the fact that the majority of subjects included in our study were non-ICU patients with stable vital signs on admission who were initially admitted to a general ward.

Nevertheless, several limitations should be considered when the results are interpreted in our study. First, as a retrospective observational study based on data collected in routine clinical care at a single tertiary center, selection bias is not to be excluded. Second, a protocol was not established for the treatment of lowing ammonia and for measuring ammonia at different time points. Third, as known, sarcopenia may influence ammonia levels and increase the risk of hepatic encephalopathy. Unfortunately, a large number of patients lack evaluation of skeletal muscle mass in our study. Hence, we are unable to assess the potential impact of sarcopenia on serum ammonia levels or patients' outcomes. Fourth, we did not explore the underlying mechanism of the contribution of ammonia level to the worse clinical outcome in HBV-ACLF patients. Hence, further prospective multi-center studies are required to elucidate the mechanism and validate the findings.

In summary, the present study highlights the importance of dynamic ammonia level measurements in predicting overt HE and mortality. Peak AMM-ULN shows the best performance as a prognostic marker in the evaluation of patients with HBV-ACLF. Patients with decreased ammonia levels suggest favorable clinical outcomes. Monitoring ammonia variation appears to help predict HBV-ACLF patients' prognosis and is useful in clinical practice's decision-making.

## Data availability statement

The raw data supporting the conclusions of this article will be made available by the authors, without undue reservation.

## Ethics statement

The studies involving humans were approved by Institute Ethics Committee of the First Affiliated Hospital of Wenzhou Medical University (KY2021-R055). The studies were conducted in accordance with the local legislation and institutional requirements. The ethics committee/institutional review board waived the requirement of written informed consent for participation from the participants or the participants' legal guardians/next of kin because the observational nature of the study.

## Author contributions

Y-JC: Data curation, Formal analysis, Investigation, Methodology, Software, Writing—original draft. J-JD: Data curation, Investigation, Methodology, Software, Writing—original draft. R-CC: Data curation, Methodology, Writing—review & editing. Q-QX: Data curation, Formal analysis, Writing—review & editing. X-ML: Data curation, Software, Writing—review & editing. D-YC: Methodology, Writing—review & editing. CC: Investigation, Writing—review & editing. X-LL: Data curation, Investigation, Writing—review & editing. K-QS: Conceptualization, Supervision, Writing—review & editing. M-QL: Conceptualization, Funding acquisition, Supervision, Writing—original draft, Writing—review & editing.

## Supplementary material

Supplementary Figure 1 Distribution of Peak AMM-ULN in HBV-ACLF patients according to the presence of overt HE **(A)** and survival **(B)**. The bottom and top of each box represent the 25th and 75th percentiles, giving the interquartile range. The horizontal bar inside the box represents the median value. AMM-ULN, ammonia level corrected to the upper limit of normal; HBV-ACLF, hepatitis B virus-related acute-on-chronic liver failure.

Supplementary Figure 2 Scatter graphs illustrating the correlations between Peak AMM-ULM and Child-Pugh, MELD, MELD-NA, CLIF-SOFA, CLIF-C ACLF, and AARC score among patients with HBV-ACLF. Peak AMM-ULM was positively correlated to the above scores. AMM-ULM, ammonia level corrected to the upper limit of normal; MELD, Model for End-Stage Liver Disease; MELD-NA, MELD-sodium; CLIF-SOFA, Chronic Liver Failure Consortium Organ Failure score; CLIF-C ACLF, CLIF-Consortium-ACLF; AARC, APASL ACLF Research Consortium; HBV-ACLF, hepatitis B virus-related acute-on-chronic liver failure.

Supplementary Figure 3 Comparison of AMM-ULM levels (Baseline, Peak, Trough) between patients with different grades of HBV-ACLF. AMM-ULN, ammonia level corrected to the upper limit of normal. HBV-ACLF, hepatitis B virus-related acute-on-chronic liver failure.

Supplementary Figure 4 Comparison of the probability of HBV-ACLF grades between patients stratifying by AMM-ULM levels cut-off values (Baseline: 1.1; Peak: 1.8; Trough: 0.9). HBV-ACLF, hepatitis B virus-related acute-on-chronic liver failure; AMM-ULN, ammonia level corrected to the upper limit of normal.

Supplementary Figure 5 Kaplan-Meier graph of 28-day survival, 3-month survival, and 1-year survival stratified by Baseline AMM-ULM <1.1 or ≥1.1. **(A-C)** Kaplan-Meier graph of 28-day survival, 3-month survival, and 1-year survival stratified by Trough AMM-ULM <0.9 or ≥ 0.9. **(D–F)** The mortality among patients with Baseline AMM-ULM ≥ 1.1 and Trough AMM-ULM ≥ 0.9 were significantly higher than those with Baseline AMM-ULM <1.1 and Trough AMM-ULM <0.9 (log-rank test, *P* < 0.001). AMM-ULN, ammonia level corrected to the upper limit of normal.

Supplementary Figure 6 ROC curves and Kaplan-Meier graphs of 28-day mortality stratified by Peak AMM-ULM <1.8 or ≥1.8 in patients with bacterial infection **(A, B)** and non-bacterial infection **(C, D)**. AMM-ULN, ammonia level corrected to the upper limit of normal; ROC, Receiver operating characteristics curve analysis.

Supplementary Figure 7 Comparison of Peak AMM-ULM levels at day 0, days 1–3, days 4–7, and day >7 between patients with HE grade 0/1 and grade 2/3/4 **(A)**, and 28-day survivors and non-survivors **(B)**. AMM-ULN, ammonia level corrected to the upper limit of normal.

Supplementary Figure 8 Probability of HE grade 2/3/4 among patients with Peak AMM-ULM levels appearing at day 0, days 1–3, days 4–7, and day >7. Among all patients **(A)** or patients with Peak AMM-ULN ≥1.8 **(B)**, patients of group (days 1–3) have a higher probability of HE grade 2/3/4 development compared to the patients of other time points groups. Among patients of group (days 1–3), there was a statistical difference in the probability of HE grade 2/3/4 between patients with Peak AMM-ULN ≥1.8 and Peak AMM-ULN <1.8 **(C)**. AMM-ULN, ammonia level corrected to the upper limit of normal.

Supplementary Figure 9 Kaplan-Meier graphs of 28-day survival, 3-month survival, and 1-year survival stratified by patients in which whether the ammonia levels decreased after the peak AMM-ULN value had been obtained. AMM-ULN, ammonia level corrected to the upper limit of normal.

Supplementary Table 1 Univariate and multivariate analysis for predictors of overt HE (grade 2/3/4).

Supplementary Table 2 Comparison of patients complications and clinical outcomes stratified by change of ammonia level (*n* = 276).

Supplementary Table 3 Comparison of HE episodes and HE reduction between groups stratified by ACLF patients in which whether the ammonia levels decreased after the peak AMM-ULN value had been obtained.
