# Supplementary figures and images for: Serum ammonia variation predicts mortality in patients with hepatitis B virus-related acute-on-chronic liver failure
Source: Front Microbiol. 2023 Dec 4;14:1282106. doi: 10.3389/fmicb.2023.1282106 (PMC10725913; doi:10.3389/fmicb.2023.1282106)

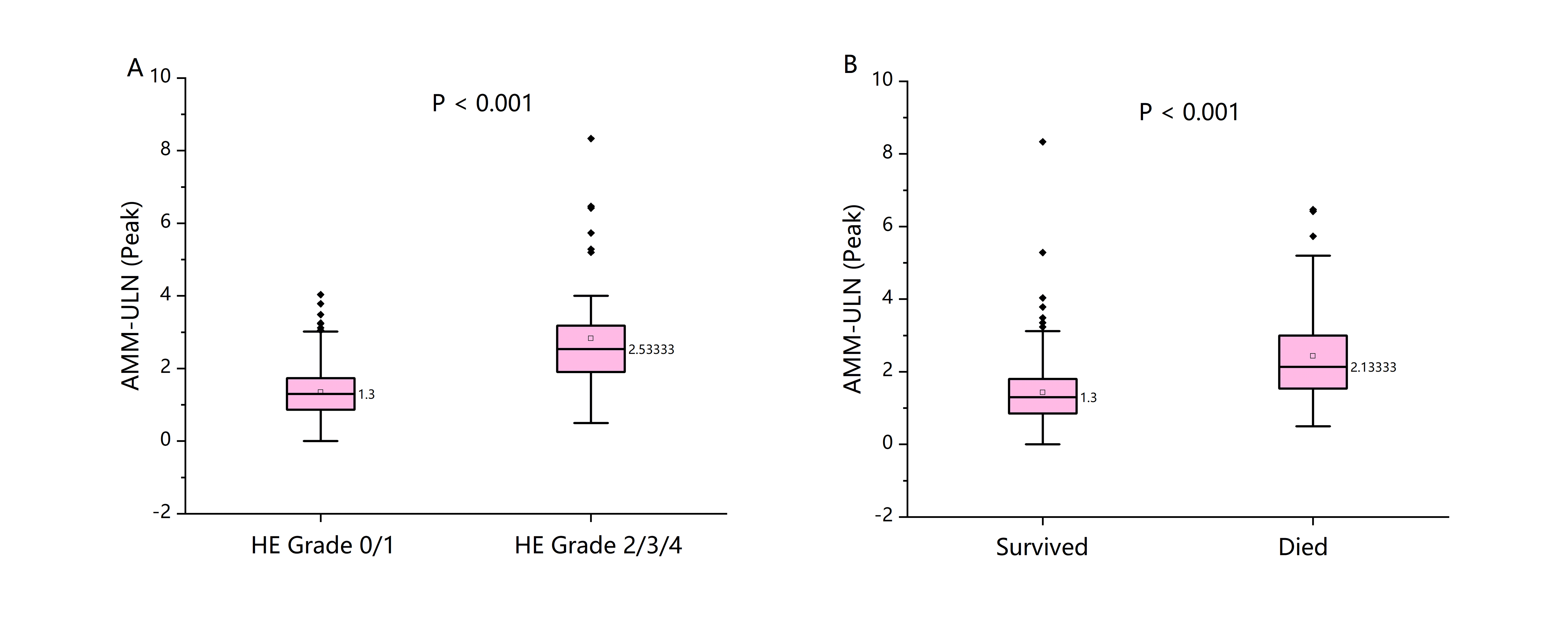

Supplement: Supplementary Figure 1 — Distribution of Peak AMM-ULN in HBV-ACLF patients according to the presence of overt HE (A) and survival (B). The bottom and top of each box represent the 25th and 75th percentiles, giving the interquartile range. The horizontal bar inside the box represents the median value. AMM-ULN, ammonia level corrected to the upper limit of normal; HBV-ACLF, hepatitis B virus-related acute-on-chronic liver failure. [file Image_1.TIF]

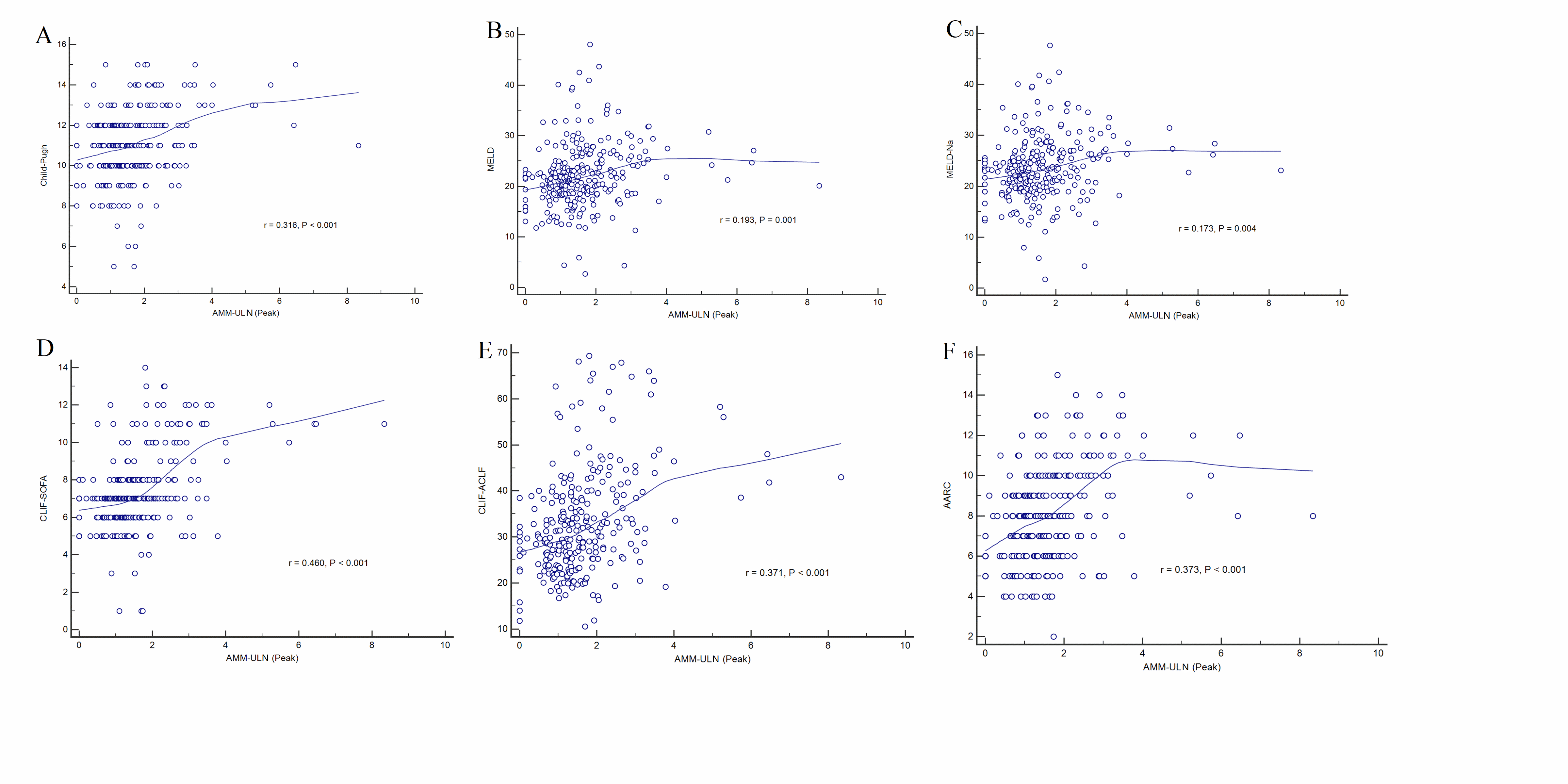

Supplement: Supplementary Figure 2 — Scatter graphs illustrating the correlations between Peak AMM-ULM and Child-Pugh, MELD, MELD-NA, CLIF-SOFA, CLIF-C ACLF, and AARC score among patients with HBV-ACLF. Peak AMM-ULM was positively correlated to the above scores. AMM-ULM, ammonia level corrected to the upper limit of normal; MELD, Model for End-Stage Liver Disease; MELD-NA, MELD-sodium; CLIF-SOFA, Chronic Liver Failure Consortium Organ Failure score; CLIF-C ACLF, CLIF-Consortium-ACLF; AARC, APASL ACLF Research Consortium; HBV-ACLF, hepatitis B virus-related acute-on-chronic liver failure. [file Image_2.TIF]

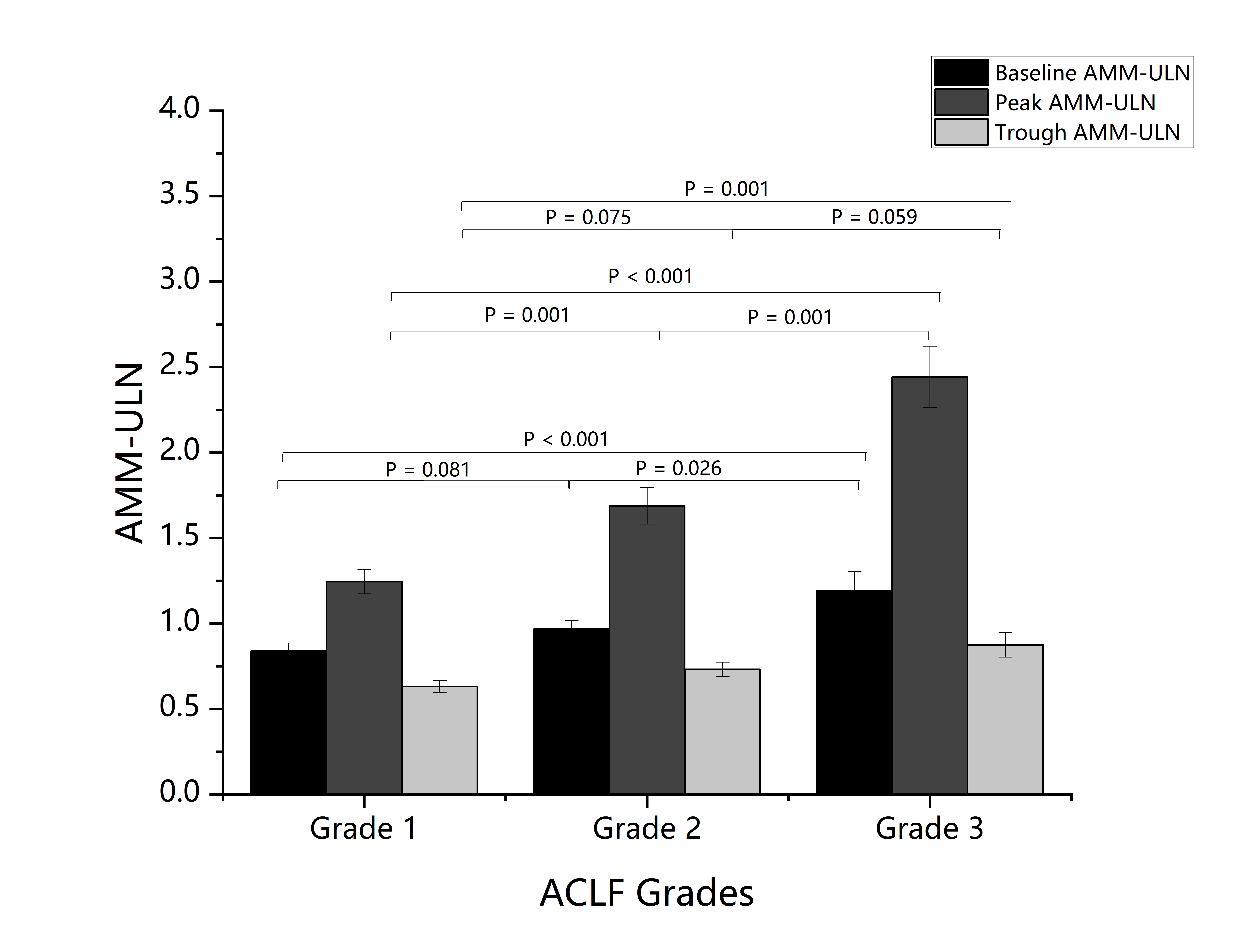

Supplement: Supplementary Figure 3 — Comparison of AMM-ULM levels (Baseline, Peak, Trough) between patients with different grades of HBV-ACLF. AMM-ULN, ammonia level corrected to the upper limit of normal. HBV-ACLF, hepatitis B virus-related acute-on-chronic liver failure. [file Image_3.TIF]

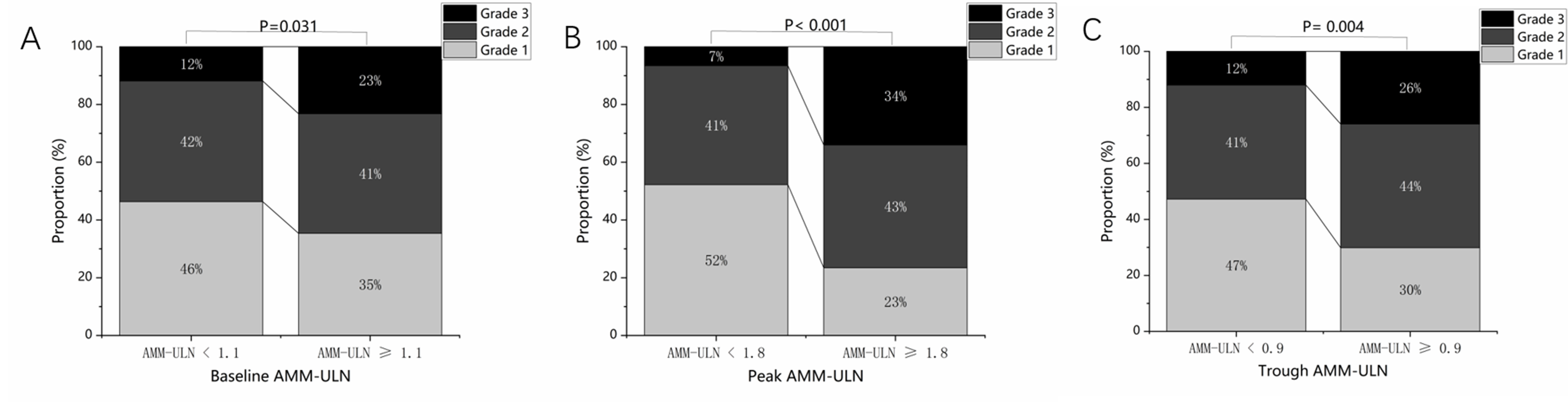

Supplement: Supplementary Figure 4 — Comparison of the probability of HBV-ACLF grades between patients stratifying by AMM-ULM levels cut-off values (Baseline: 1.1; Peak: 1.8; Trough: 0.9). HBV-ACLF, hepatitis B virus-related acute-on-chronic liver failure; AMM-ULN, ammonia level corrected to the upper limit of normal. [file Image_4.TIF]

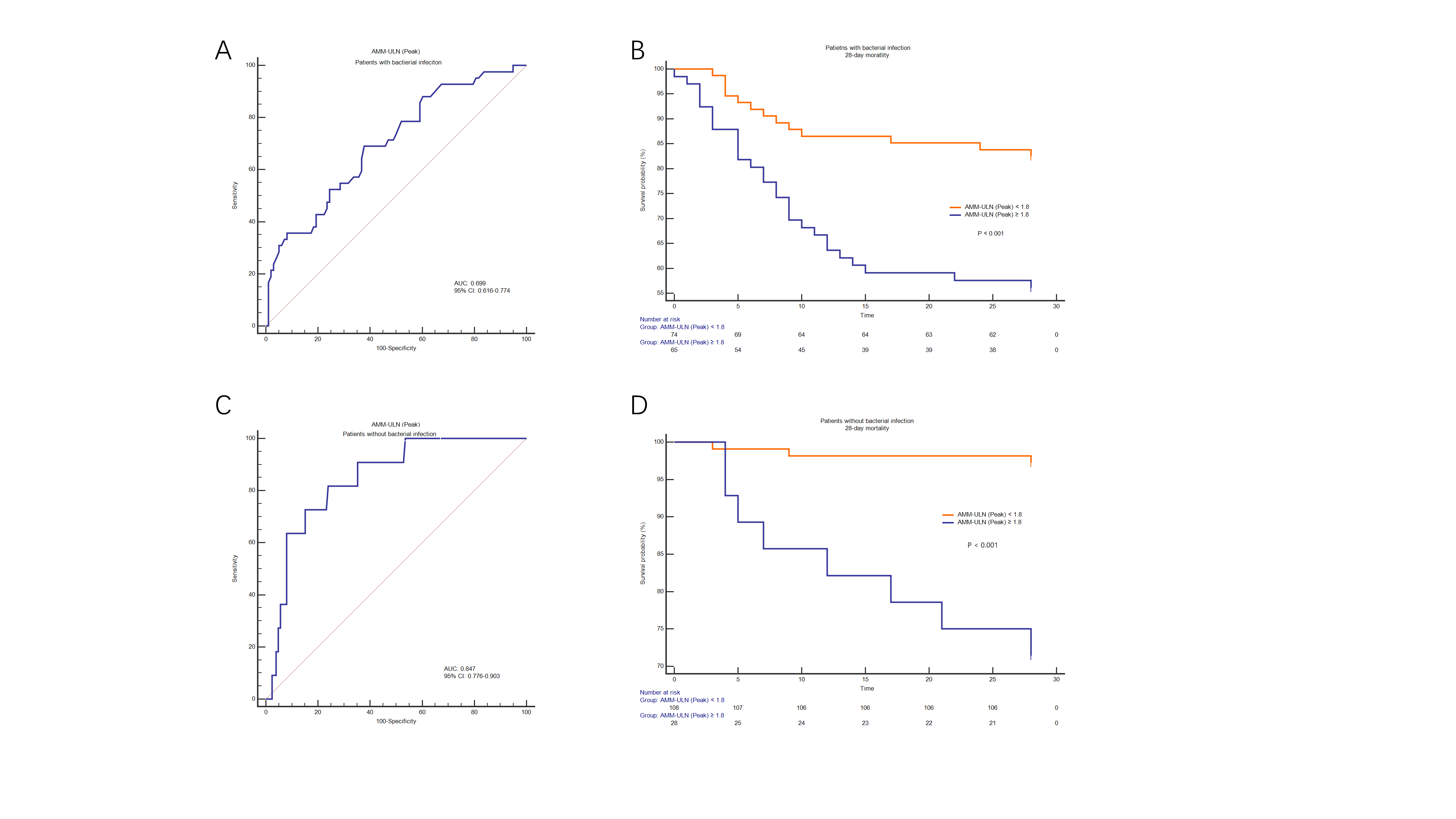

Supplement: Supplementary Figure 6 — ROC curves and Kaplan-Meier graphs of 28-day mortality stratified by Peak AMM-ULM <1.8 or ≥1.8 in patients with bacterial infection (A, B) and non-bacterial infection (C, D). AMM-ULN, ammonia level corrected to the upper limit of normal; ROC, Receiver operating characteristics curve analysis. [file Image_6.TIF]

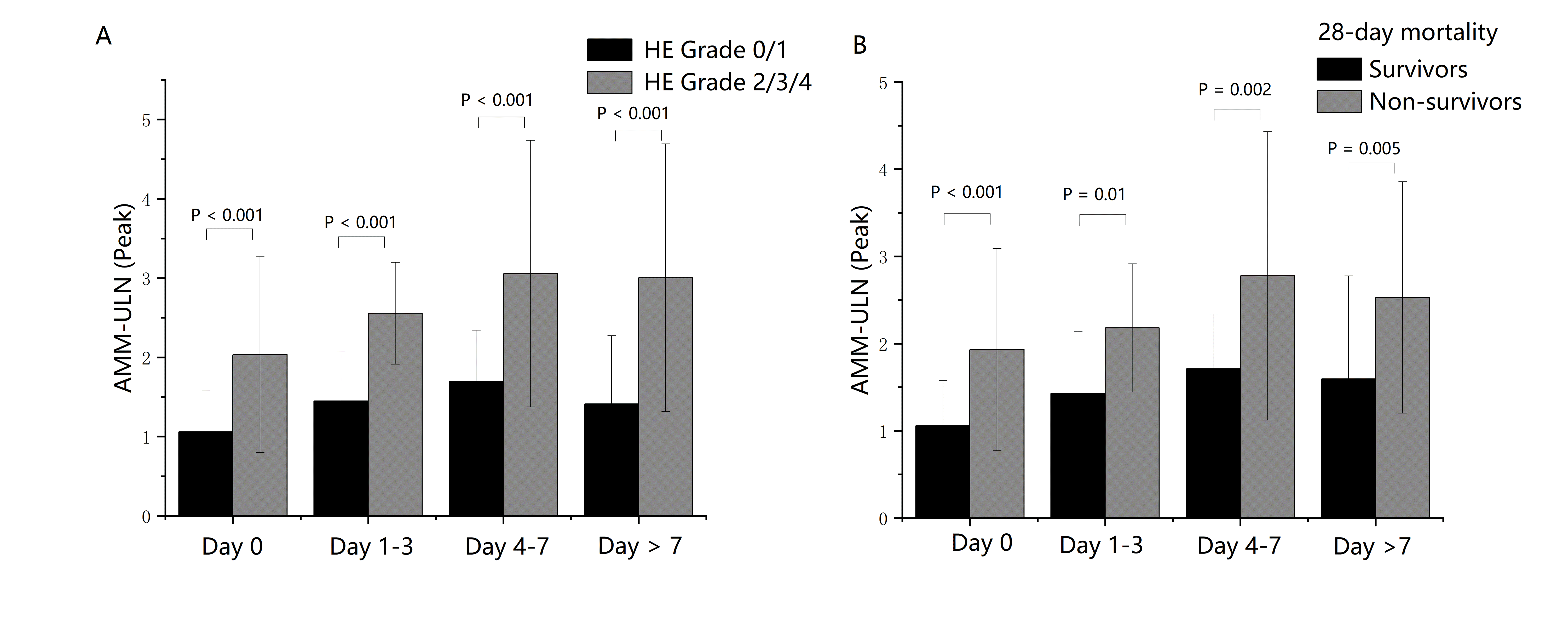

Supplement: Supplementary Figure 7 — Comparison of Peak AMM-ULM levels at day 0, days 1–3, days 4–7, and day >7 between patients with HE grade 0/1 and grade 2/3/4 (A), and 28-day survivors and non-survivors (B). AMM-ULN, ammonia level corrected to the upper limit of normal. [file Image_7.TIF]

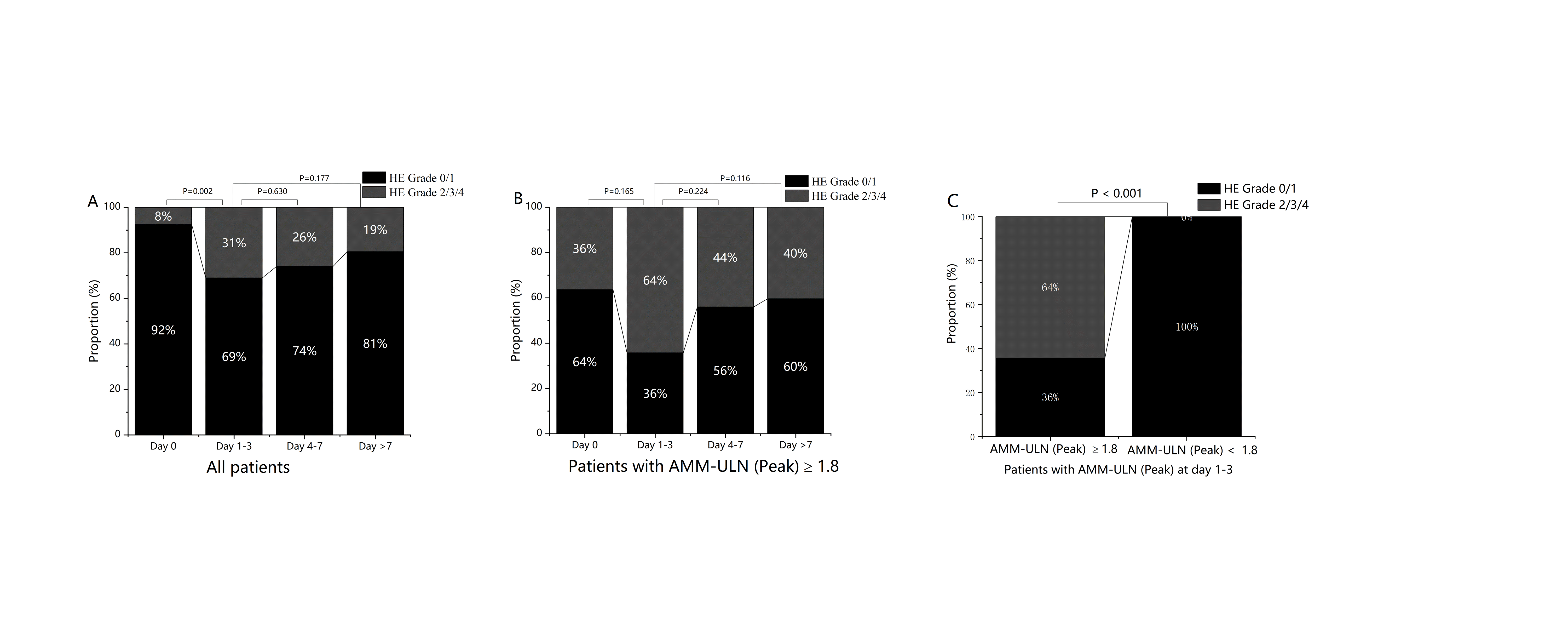

Supplement: Supplementary Figure 8 — Probability of HE grade 2/3/4 among patients with Peak AMM-ULM levels appearing at day 0, days 1–3, days 4–7, and day >7. Among all patients (A) or patients with Peak AMM-ULN ≥1.8 (B), patients of group (days 1–3) have a higher probability of HE grade 2/3/4 development compared to the patients of other time points groups. Among patients of group (days 1–3), there was a statistical difference in the probability of HE grade 2/3/4 between patients with Peak AMM-ULN ≥1.8 and Peak AMM-ULN <1.8 (C). AMM-ULN, ammonia level corrected to the upper limit of normal. [file Image_8.TIF]

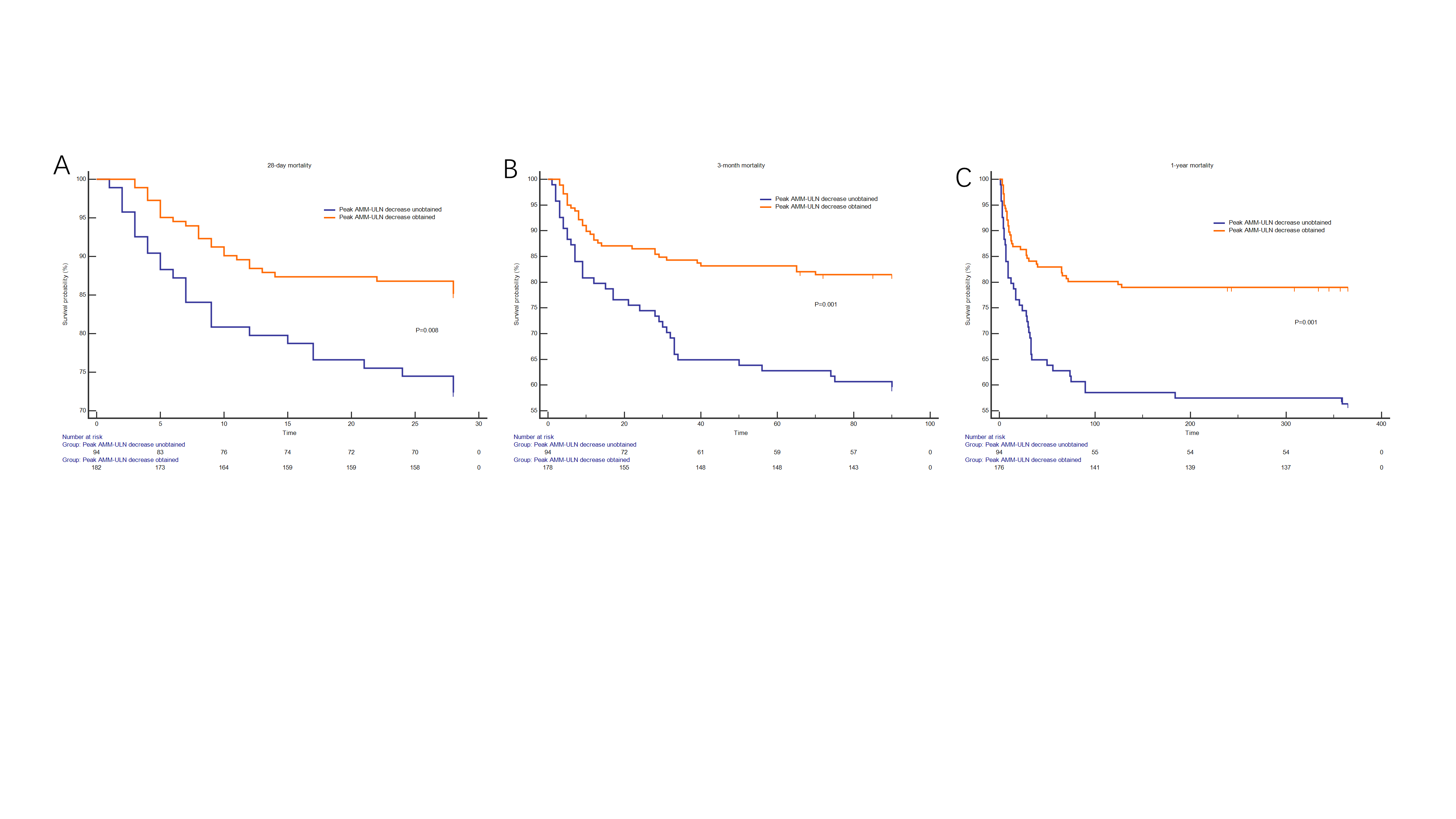

Supplement: Supplementary Figure 9 — Kaplan-Meier graphs of 28-day survival, 3-month survival, and 1-year survival stratified by patients in which whether the ammonia levels decreased after the peak AMM-ULN value had been obtained. AMM-ULN, ammonia level corrected to the upper limit of normal. [file Image_9.TIF]
