# Supplementary material for: Serum ammonia variation predicts mortality in patients with hepatitis B virus-related acute-on-chronic liver failure
Source: Front Microbiol. 2023 Dec 4;14:1282106. doi: 10.3389/fmicb.2023.1282106 (PMC10725913; doi:10.3389/fmicb.2023.1282106)
Supplement: Supplementary Table 1 — Univariate and multivariate analysis for predictors of overt HE (grade 2/3/4). [file Table_1.docx]

**Supplement table 1. Univariate and multivariate analysis for predictors of overt HE (grade 2/3/4).**

| Baseline Characteristic | Univariate Model | | Multivariate Model 1(All Significant  Continuous Variables) | | Multivariate Model 2 (Includes All  Organ Failures) | |
| --- | --- | --- | --- | --- | --- | --- |
|  | HR | P | HR | P | HR | P |
| Age (years) | **1.030(1.006-1.054)** | **0.013** | **1.041(1.002-1.081)** | **0.040** |  |  |
| Gender (male/female) | 1.042(0.432-2.515) | 0.927 |  |  |  |  |
| Diabetes mellitus | **2.462(1.141-5.311)** | **0.022** |  |  |  |  |
| Hypertension | 1.508(0.663-3.429) | 0.327 |  |  |  |  |
| Drug discontinuance | 0.812(0.369-1.785) | 0.812 |  |  |  |  |
| Alcohol | 1.790(0.850-3.771) | 0.126 |  |  |  |  |
| Cirrhosis | **2.250(1.215-4.165)** | **0.010** |  |  |  |  |
| Laboratory values |  |  |  |  |  |  |
| WBC | **1.116(1.036-1.201)** | **0.004** |  |  |  |  |
| Platelets | **0.993(0.987-1.000)** | **0.034** |  |  |  |  |
| Bilirubin (mg/dL) | **1.043(1.004-1.084)** | **0.030** |  |  |  |  |
| Albumin (g/L) | 0.969(0.915-1.026) | 0.280 |  |  |  |  |
| ALT (U/L) | 1.000(0.999-1.000) | 0.335 |  |  |  |  |
| AST(U/L) | 1.000(0.999-1.000) | 0.550 |  |  |  |  |
| INR | **3.573(2.313-5.518)** | **<0.001** | **1.258(1.258-3.902)** | **0.006** |  |  |
| PTA（%） | **0.899(0.866-0.934)** | **<0.001** |  |  |  |  |
| Creatinine (mg/dL) | 1.768(0.970-3.225) | 0.063 |  |  |  |  |
| Sodium (mmol/L) | **0.924(0.859-0.994)** | **0.034** |  |  |  |  |
| Lactate (mmol/L) | **1.238(1.050-1.461)** | **0.011** |  |  |  |  |
| AFP | **0.997(0.995-1.000)** | **0.048** |  |  |  |  |
| AMM-ULN (Baseline) | **1.017(1.007-1.026)** | **<0.001** |  |  |  |  |
| AMM-ULN (Peak) | **1.026(1.018-1.035)** | **<0.001** | **1.028(1.017-1.040)** | **<0.001** | **1.031(1.017-1.044)** | **<0.001** |
| AMM-ULN (Valley) | **1.026(1.013-1.039)** | **<0.001** |  |  |  |  |
| Bacterial infections | **3.990(1.985-8.021)** | **<0.001** |  |  |  |  |
| Upper GI bleeding | **20.023(6.898-58.123)** | **<0.001** |  |  | **9.015(1.678-48.426)** | **0.010** |
| Ascites | **1.901(0.985-3.669)** | **0.056** |  |  |  |  |
| Respiratory failure | **41.625(11.606-149.292)** | **<0.001** |  |  | **23.707(3.562-157.788)** | **0.001** |
| Circulation failure | **16.043(6.184-41.620)** | **<0.001** |  |  |  |  |
| Kidney failure | **13.476(4.033-45.031)** | **<0.001** |  |  |  |  |

Bold values represent statistical significance.

Abbreviations: WBC, white blood cell counts; ALT, alanine aminotransferase; AST, aspartate aminotransferase; INR, international normalized ratio; PTA, prothrombin activity; AFP, alpha-fetoprotein; AMM-ULN, ammonia level corrected to the upper limit of normal; GI, gastrointestinal; ACLF, acute-on-chronic liver failure.
