# Supplementary material for: Serum ammonia variation predicts mortality in patients with hepatitis B virus-related acute-on-chronic liver failure
Source: Front Microbiol. 2023 Dec 4;14:1282106. doi: 10.3389/fmicb.2023.1282106 (PMC10725913; doi:10.3389/fmicb.2023.1282106)
Supplement: Supplementary Table 2 — Comparison of patients complications and clinical outcomes stratified by change of ammonia level (n = 276). [file Table_2.docx]

**Supplement table 2. Comparison of patients complications and clinical outcomes stratified by change of ammonia level（n=276）.**

|  | Ammonia increased  N=115 | Ammonia decreased  N=161 | P |
| --- | --- | --- | --- |
| HE (grade 3/4) | 32 (27.8%) | 45 (8.1%) | **<0.001** |
| Bacterial infections | 73 (63.4%) | 67 (41.6%) | **<0.001** |
| Upper GI bleeding | 14 (12.2%) | 7 (4.3%) | **0.015** |
| Ascites (0/1) | 76 (66.1%) | 128 (79.1%) | **0.001** |
| Ascites (2/3) | 29 (33.9%) | 33 (20.9%) |  |
| Respiratory failure | 14 (12.2%) | 7 (4.3%) | **0.015** |
| Circulation failure | 16 (13.9%) | 8 (4.9%) | **0.009** |
| Kidney failure | 10 (8.7%) | 4 (2.5%) | **0.026** |
| Scores |  |  |  |
| Child-Pugh | 12 (10-13) | 10 (10-12) | **<0.001** |
| MELD | 23 (18-27) | 21 (18-23) | **0.01** |
| MELD-NA | 25 (20-29) | 22 (19-24) | **<0.001** |
| CLIF-SOFA | 7 (6-10) | 7 (6-8) | **<0.001** |
| CLIF-C-ACLF | 34.9 (27.7-44.6) | 28.4 (23.4-34.0) | **<0.001** |
| AARC | 9 (7-11) | 7 (6-9) | **<0.001** |
| ACLF |  |  |  |
| Grade I | 33 (28.6%) | 84 (52.2%) | **<0.001** |
| Grade II | 50 (43.5%) | 65 (40.4%) |  |
| Grade III | 32 (27.9%) | 12 (7.4%) |  |
| 28-day mortality (%) | 34 (29.6%) | 19 (11.8%) | **<0.001** |
| 3-month mortality (%) | 47 (40.8%) | 24 (14.9%) | **<0.001** |
| 1-year mortality (%) | 53 (46.8%) | 25 (15.5%) | **<0.001** |

Bold values represent statistical significance.

Abbreviations: HE, hepatic encephalopathy; GI, gastrointestinal; ACLF, acute-on-chronic liver failure.
