# Supplementary material for: Serum ammonia variation predicts mortality in patients with hepatitis B virus-related acute-on-chronic liver failure
Source: Front Microbiol. 2023 Dec 4;14:1282106. doi: 10.3389/fmicb.2023.1282106 (PMC10725913; doi:10.3389/fmicb.2023.1282106)
Supplement: Supplementary Table 3 — Comparison of HE episodes and HE reduction between groups stratified by ACLF patients in which whether the ammonia levels decreased after the peak AMM-ULN value had been obtained. [file Table_3.docx]

**Supplement table 3. Comparison of HE episodes and HE reduction between groups stratified by ACLF patients in which whether the ammonia levels decreased after the peak AMM-ULN value had been obtained.**

|  | Patients with Peak AMM-ULN decrease obtained  N=182 | Patients with Peak AMM-ULN decrease un-obtained  N=94 | P |
| --- | --- | --- | --- |
| HE | 35 (19.2%) | 29 (30.8%) | **P= 0.030** |
| HE reduction | 13 (37.2%) | 3 (10.3%) | **P=0.014** |

Bold values represent statistical significance.

Abbreviations: HE, hepatic encephalopathy; ACLF, acute-on-chronic liver failure. AMM-ULN, ammonia level corrected to the upper limit of normal.
