# Supplementary material for: Serum ammonia variation predicts mortality in patients with hepatitis B virus-related acute-on-chronic liver failure
Source: Front Microbiol. 2023 Dec 4;14:1282106. doi: 10.3389/fmicb.2023.1282106 (PMC10725913; doi:10.3389/fmicb.2023.1282106)
Supplement: Supplementary file 13 [file Data_Sheet_1.docx]

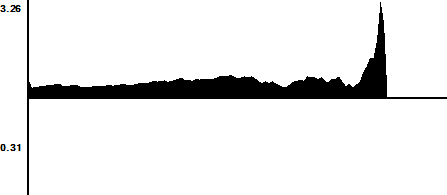
Survival Analysis: AMM-ULM (Baseline)

April 2, 2023 10:28:06

caiyijing007


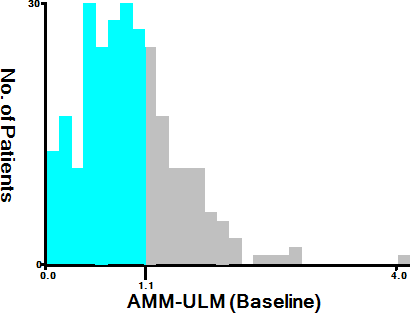


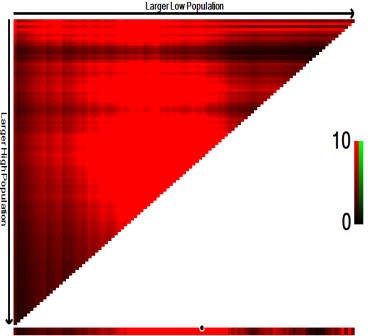


**Chi-sq Hi/Lo**


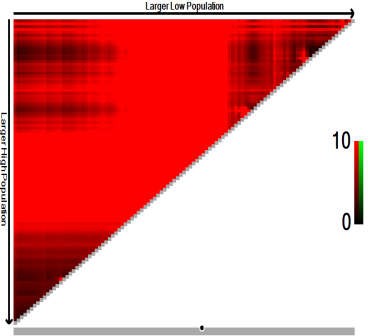


**Chi-Sq Hi/Mid/Lo**


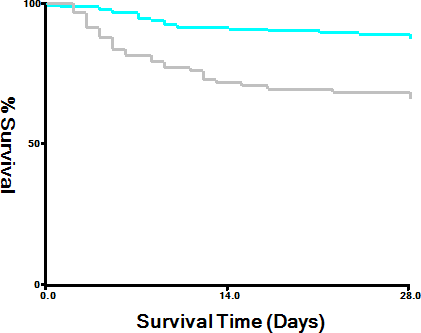
**Subpopulation Cutpoints:**

| Pt No | % Total | Events | Rate | Rank | Range |
| --- | --- | --- | --- | --- | --- |
| 184 | 66.67 | 22 | 11.96 | 0 to 58 | 0.00 thru 1.12 |
| 92 | 33.33 | 31 | 33.70 | 59 to 105 | 1.13 thru 4.00 |
| 276 | 100.00 | 53 | 19.20 | 0 to 105 | 0.00 thru 4.00 |
| **Statistics:** | |  | |  | |
| Variable | | Value | |  | |
| Miller-Seigmund P | | 0.0005 | | Max: 0.0005 | |
| Chi-sq Hi/Lo | | 20.0534 | | Max: 20.0534 | |
| Relative Risk 1 vs 2 | | 1.00 / 2.82 | |  | |


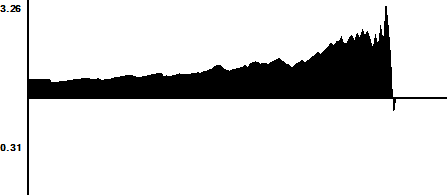
Survival Analysis: AMM-ULM (Peak)

April 2, 2023 10:23:45

caiyijing007


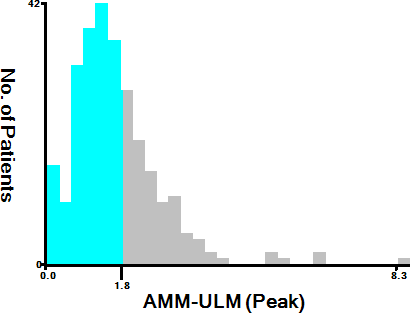


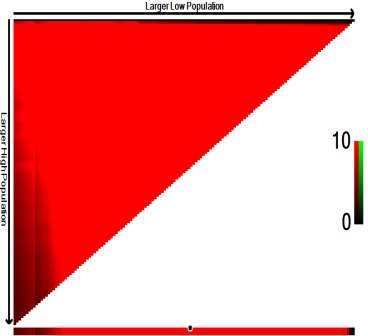


**Chi-sq Hi/Lo**


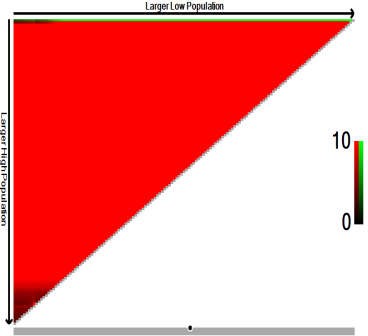


**Chi-Sq Hi/Mid/Lo**


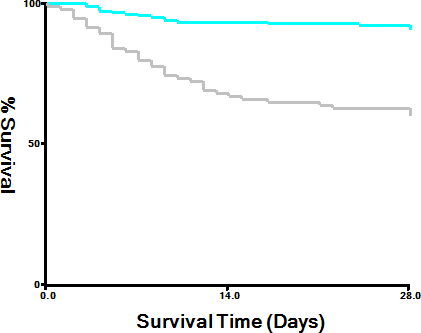
**Subpopulation Cutpoints:**

| Pt No | % Total | Events | Rate | Rank | Range |
| --- | --- | --- | --- | --- | --- |
| 182 | 65.94 | 16 | 8.79 | 0 to 73 | 0.00 thru 1.77 |
| 94 | 34.06 | 37 | 39.36 | 74 to 141 | 1.80 thru 8.33 |
| 276 | 100.00 | 53 | 19.20 | 0 to 141 | 0.00 thru 8.33 |
| **Statistics:** | |  | |  | |
| Variable | | Value | |  | |
| Miller-Seigmund P | | <0.0001 | | Max: <0.0001 | |
| Chi-sq Hi/Lo | | 40.1488 | | Max: 40.1488 | |
| Relative Risk 1 vs 2 | | 1.00 / 4.48 | |  | |


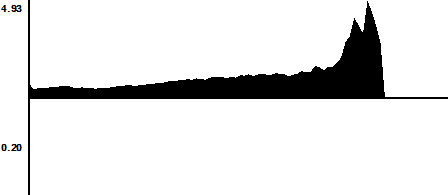
Survival Analysis: NH3-ULM (valley)

April 2, 2023 10:33:06

caiyijing007


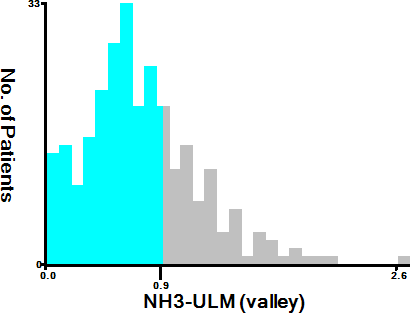


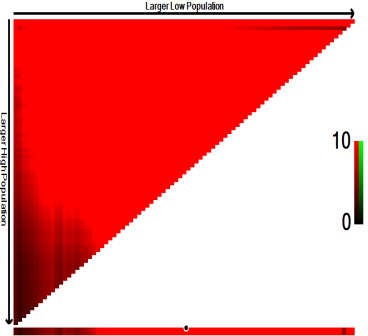


**Chi-sq Hi/Lo**


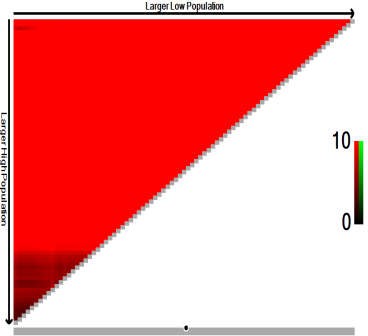


**Chi-Sq Hi/Mid/Lo**


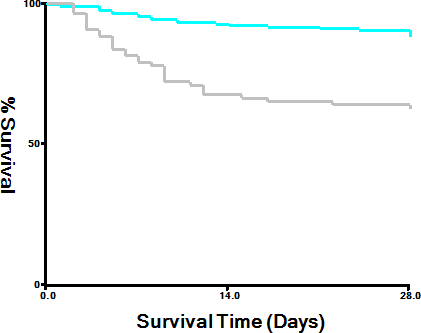
**Subpopulation Cutpoints:**

| Pt No | % Total | Events | Rate | Rank | Range |
| --- | --- | --- | --- | --- | --- |
| 190 | 68.84 | 21 | 11.05 | 0 to 42 | 0.00 thru 0.85 |
| 86 | 31.16 | 32 | 37.21 | 43 to 83 | 0.87 thru 2.63 |
| 276 | 100.00 | 53 | 19.20 | 0 to 83 | 0.00 thru 2.63 |
| **Statistics:** | |  | |  | |
| Variable | | Value | |  | |
| Miller-Seigmund P | | <0.0001 | | Max: <0.0001 | |
| Chi-sq Hi/Lo | | 28.8458 | | Max: 28.8458 | |
| Relative Risk 1 vs 2 | | 1.00 / 3.37 | |  | |
